# Supplementary material for: Iterative improvement in the automatic modular design of robot swarms
Source: PeerJ Comput Sci. 2020 Dec 7;6:e322. doi: 10.7717/peerj-cs.322 (PMC7924708; doi:10.7717/peerj-cs.322)
Supplement: Supplemental Information 3 [file peerj-cs-06-322-s003.zip › argos3/doc/api/standalone/a00510_source.html]

ARGoS: plugins/robots/generic/control\_interface/ci\_quadrotor\_position\_actuator.cpp Source File


- Main Page
- Related Pages
- Namespaces
- Classes
- Files

- File List
- File Members

# plugins/robots/generic/control\_interface/ci\_quadrotor\_position\_actuator.cpp

Go to the documentation of this file.

```
00001 
00007 #include "ci_quadrotor_position_actuator.h"
00008 
00009 #ifdef ARGOS_WITH_LUA
00010 #include <argos3/core/wrappers/lua/lua_utility.h>
00011 #endif
00012 
00013 namespace argos {
00014 
00015    /****************************************/
00016    /****************************************/
00017 
00018 #ifdef ARGOS_WITH_LUA
00019    /*
00020     * The stack must have three values in this order:
00021     * 1. x coordinate (a number)
00022     * 2. y coordinate (a number)
00023     * 3. z coordinate (a number)
00024     */
00025    int LuaSetAbsolutePosition(lua_State* pt_lua_state) {
00026       /* Check parameters */
00027       if(lua_gettop(pt_lua_state) != 3) {
00028          return luaL_error(pt_lua_state, "robot.quadrotor.set_absolute_position() expects 3 arguments");
00029       }
00030       luaL_checktype(pt_lua_state, 1, LUA_TNUMBER);
00031       luaL_checktype(pt_lua_state, 2, LUA_TNUMBER);
00032       luaL_checktype(pt_lua_state, 3, LUA_TNUMBER);
00033       /* Perform action */
00034       CLuaUtility::GetDeviceInstance<CCI_QuadRotorPositionActuator>(pt_lua_state, "quadrotor")->
00035          SetAbsolutePosition(CVector3(lua_tonumber(pt_lua_state, 1),
00036                                       lua_tonumber(pt_lua_state, 2),
00037                                       lua_tonumber(pt_lua_state, 3)));
00038       return 0;
00039    }
00040 #endif
00041 
00042    /****************************************/
00043    /****************************************/
00044 
00045 #ifdef ARGOS_WITH_LUA
00046    /*
00047     * The stack must have three values in this order:
00048     * 1. x coordinate (a number)
00049     * 2. y coordinate (a number)
00050     * 3. z coordinate (a number)
00051     */
00052    int LuaSetRelativePosition(lua_State* pt_lua_state) {
00053       /* Check parameters */
00054       if(lua_gettop(pt_lua_state) != 3) {
00055          return luaL_error(pt_lua_state, "robot.quadrotor.set_relative_position() expects 3 arguments");
00056       }
00057       luaL_checktype(pt_lua_state, 1, LUA_TNUMBER);
00058       luaL_checktype(pt_lua_state, 2, LUA_TNUMBER);
00059       luaL_checktype(pt_lua_state, 3, LUA_TNUMBER);
00060       /* Perform action */
00061       CLuaUtility::GetDeviceInstance<CCI_QuadRotorPositionActuator>(pt_lua_state, "quadrotor")->
00062          SetRelativePosition(CVector3(lua_tonumber(pt_lua_state, 1),
00063                                       lua_tonumber(pt_lua_state, 2),
00064                                       lua_tonumber(pt_lua_state, 3)));
00065       return 0;
00066    }
00067 #endif
00068 
00069    /****************************************/
00070    /****************************************/
00071 
00072 #ifdef ARGOS_WITH_LUA
00073    /*
00074     * The stack must have one value:
00075     * 1. yaw angle (in radians)
00076     */
00077    int LuaSetAbsoluteYaw(lua_State* pt_lua_state) {
00078       /* Check parameters */
00079       if(lua_gettop(pt_lua_state) != 1) {
00080          return luaL_error(pt_lua_state, "robot.quadrotor.set_absolute_yaw() expects 1 argument");
00081       }
00082       luaL_checktype(pt_lua_state, 1, LUA_TNUMBER);
00083       /* Perform action */
00084       CLuaUtility::GetDeviceInstance<CCI_QuadRotorPositionActuator>(pt_lua_state, "quadrotor")->
00085          SetAbsoluteYaw(CRadians(lua_tonumber(pt_lua_state, 1) / 180.0f * CRadians::PI));
00086       return 0;
00087    }
00088 #endif
00089 
00090    /****************************************/
00091    /****************************************/
00092 
00093 #ifdef ARGOS_WITH_LUA
00094    /*
00095     * The stack must have one value:
00096     * 1. yaw angle (in radians)
00097     */
00098    int LuaSetRelativeYaw(lua_State* pt_lua_state) {
00099       /* Check parameters */
00100       if(lua_gettop(pt_lua_state) != 1) {
00101          return luaL_error(pt_lua_state, "robot.quadrotor.set_relative_yaw() expects 1 argument");
00102       }
00103       luaL_checktype(pt_lua_state, 1, LUA_TNUMBER);
00104       /* Perform action */
00105       CLuaUtility::GetDeviceInstance<CCI_QuadRotorPositionActuator>(pt_lua_state, "quadrotor")->
00106          SetRelativeYaw(CRadians(lua_tonumber(pt_lua_state, 1) / 180.0f * CRadians::PI));
00107       return 0;
00108    }
00109 #endif
00110 
00111    /****************************************/
00112    /****************************************/
00113 
00114 #ifdef ARGOS_WITH_LUA
00115    void CCI_QuadRotorPositionActuator::CreateLuaState(lua_State* pt_lua_state) {
00116       CLuaUtility::OpenRobotStateTable(pt_lua_state, "quadrotor");
00117       CLuaUtility::AddToTable(pt_lua_state, "_instance", this);
00118       CLuaUtility::AddToTable(pt_lua_state, "set_absolute_position", &LuaSetAbsolutePosition);
00119       CLuaUtility::AddToTable(pt_lua_state, "set_absolute_yaw", &LuaSetAbsoluteYaw);
00120       CLuaUtility::AddToTable(pt_lua_state, "set_relative_position", &LuaSetRelativePosition);
00121       CLuaUtility::AddToTable(pt_lua_state, "set_relative_yaw", &LuaSetRelativeYaw);
00122       CLuaUtility::CloseRobotStateTable(pt_lua_state);
00123    }
00124 #endif
00125 
00126    /****************************************/
00127    /****************************************/
00128 
00129 }
```

---

Generated on 10 Jul 2018 for ARGoS by 
 1.6.1
